# Atomistic Mechanism of MicroRNA Translation Upregulation via Molecular Dynamics Simulations

**DOI:** 10.1371/journal.pone.0043788

**Published:** 2012-08-27

**Authors:** Wei Ye, Fang Qin, Jian Zhang, Ray Luo, Hai-Feng Chen

**Affiliations:** 1 State Key Laboratory of Microbial metabolism, Department of Bioinformatics and Biostatistics, College of Life Sciences and Biotechnology, Shanghai Jiaotong University, Shanghai, China; 2 Department of Pathophysiology, Key Laboratory of Cell Differentiation and Apoptosis of Chinese Ministry of Education, School of Medicine, Shanghai Jiaotong University, Shanghai, China; 3 Department of Molecular Biology and Biochemistry, University of California Irvine, Irvine, California, United States of America; 4 Department of Computational Biology, Shanghai Center for Bioinformation Technology, Shanghai, China; Semmelweis University, Hungary

## Abstract

MicroRNAs are endogenous 23–25 nt RNAs that play important gene-regulatory roles in animals and plants. Recently, miR369-3 was found to upregulate translation of TNFα mRNA in quiescent (G0) mammalian cell lines. Knock down and immunofluorescence experiments suggest that microRNA-protein complexes (with FXR1 and AGO2) are necessary for the translation upregulation. However the molecular mechanism of microRNA translation activation is poorly understood. In this study we constructed the microRNA-mRNA-AGO2-FXR1 quadruple complex by bioinformatics and molecular modeling, followed with all atom molecular dynamics simulations in explicit solvent to investigate the interaction mechanisms for the complex. A combined analysis of experimental and computational data suggests that AGO2-FXR1 complex relocalize microRNA:mRNA duplex to polysomes in G0. The two strands of dsRNA are then separated upon binding of AGO2 and FXR1. Finally, polysomes may improve the translation efficiency of mRNA. The mutation research confirms the stability of microRNA-mRNA-FXR1 and illustrates importance of key residue of Ile304. This possible mechanism can shed more light on the microRNA-dependent upregulation of translation.

## Introduction

MicroRNAs are endogenous 23–25 nucleotide RNAs that play important gene-regulatory roles in animals and plants by pairing to the mRNAs of protein-coding genes to direct their posttranscriptional regulation. [1] These small RNAs recognize 3′ untranslated regions (3′ UTR) of target mRNAs through complementary base pairing, recruit RNA-induced silencing complex to the target mRNA, and repress the translation of mRNA in cycling/proliferating cells. [2] Argonaute 2 (AGO2) is the core component of microRNA ribonucleoprotein complex (miRNP). It consists of four subunits: PAZ, PIWI, and two terminal domains. [3] PAZ and PIWI domains bind 3′ terminal nucleotide and 5′ terminal nucleotide, respectively, anchoring the single strand microRNA into the groove. [4], [5], [6] MicroRNAs recognize and bind seed sequences in the 3′ UTR of target mRNA. Furthermore, miRNP can be located to the target mRNA and recruit different regulatory factors such as GW182 and FXR1 to regulate the translation process. Among these factors, FXR1 and AGO2 have received more attentions due to their roles in microRNA regulation and human diseases. [7] Recently, it is found that miR369-3 upregulates the translation of TNFα mRNA in quiescent cell under serum starvation. [8] That is, once the cell enters into the stage of quiescence, microRNA up-regulates the translation of target mRNAs. As for siRNA, the target gene is silenced whether the cell enters into the quiescent state. [9] This indicates that the status of base pair plays a key role in the biological function of small RNAs. Knock down and immunofluorescence experiments indicate that microRNA-protein complexes (with FXR1 and AGO2) are necessary for the translation upregulation. [10], [11].

FXR1 has seven spliced and conserved isoforms in mammals. [12], [13] Experimental observations also demonstrate that isoform a of FXR1 can interact with AGO2 directly or indirectly in the microRNA pathway. [10], [14], [15] FXR1 helps assemble one strand of the miRNA:mRNA* selectively into the hydrophobic groove of AGO2, which is carried out by the KH domain of FXR1. As a nucleic acid chaperone, [16] KH domain binds to nucleic acid molecules and prevents them from folding into disordered form. The finding supports the conclusion that FXR1 assembles microRNAs into AGO2. [17] Therefore, identification of the protein components of the RNA-associated complex is one of the key steps to study up-regulation translation of microRNA. [8], [14] However without any atomic structure of the complex, detailed upregulation mechanism is poorly understood. To study the atomic mechanism of translation upregulation for microRNA, we modelled their atomic structures with bioinformatics methods. These atomic-resolution structures make it possible to investigate the upregulation mechanism and compare with experimental observations directly.

**Figure 1:**
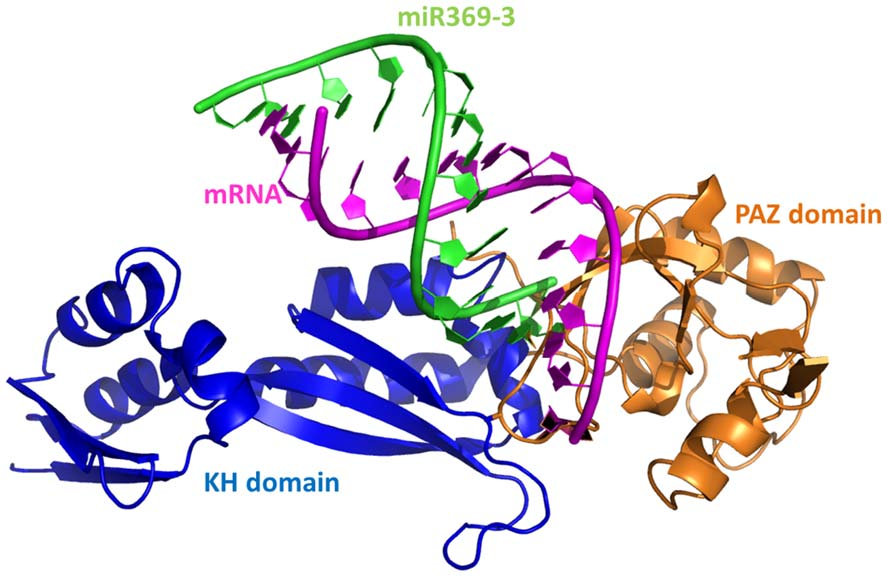
The quadruple complex for PAZ-dsRNA-KH. Major secondary structures are indicated. PAZ represents in orange, KH domain in blue, mRNA in magenta, miR369-3 in green.

**Table 1:** Summary of simulation conditions.

Compound	counter ion	water	time(ns)
dsRNA	18 Na+	5232	50
PAZ-dsRNA	11 Na+	11608	60
KH-dsRNA	30 Na+	11953	50
Mutant KH-dsRNA	30 Na+	11958	50
PAZ-dsRNA-KH complex	15 Na+	26791	50

As reviewed, previous experimental efforts provide direct evidence for the translation activation function of microRNA with the interdependence of both FXR1 and AGO2. [14] In this study we focus on the specific recognition between dsRNA and FXR1-AGO2. Our research intends to address two interesting questions: (a) conformational change of dsRNA upon binding of FXR1-AGO2, and (b) the molecular mechanism in the recognition between dsRNA and FXR1-AGO2. To shed light on these questions, we carried out multiple molecular dynamics simulations in explicit water to study the binding mode and to infer the possible upregulation pathway.

## Results

### 1. Stability of Solvated Systems

As reviewed, dsRNA/AGO2 interaction induces gene silencing. Under serum-starvation conditions, FXR1 recruits AGO2 and dsRNA to facilitate the interaction. Furthermore, experimental evidence supports the translation activation function of both FXR1 and AGO2 and demonstrates their inter-dependence for upregulation. Therefore, analysis of different complexation modes among dsRNA, AGO2, and FXR1 is the first step to understand the upregulation mechanism of microRNA.

**Figure 2:**
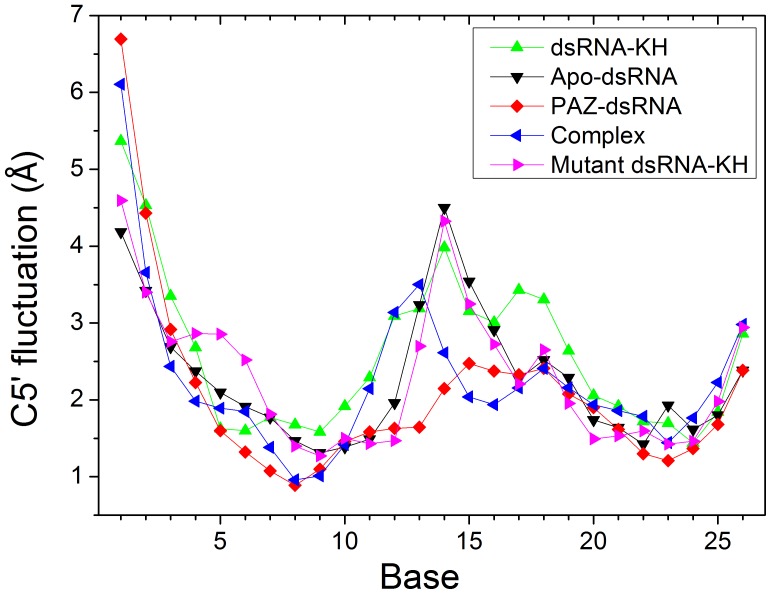
C5’ variation of holo and apo states for dsRNA in five systems.

Thus four complexation combinations of dsRNA for miRNA369-3 and mRNA with PAZ, KH domain, PAZ-KH were modeled. Specifically apo-dsRNA, dsRNA-PAZ, dsRNA-KH, and dsRNA-PAZ-KH were studied in this work. The structure of quadruple complex (dsRNA-PAZ-KH) is shown in Figure 1. Because experimental observation suggests that Ile304 of KH is the critical residue and Ile304Asn mutation leads to loss of secondary structure in the KH domain, [18], [19], [20] dsRNA with mutant KH was also simulated. The stability of these solvated systems was first analyzed with explicit solvent molecular dynamics at 298 K. The simulation conditions are listed in Table 1. To confirm the equilibration of the tested systems, the C5’ RMSDs relative to the average structure of holo- and apo-dsRNA are illustrated in Figure S1 of Supplementary Materials. The figure shows that apo-dsRNA, PAZ-dsRNA, KH-dsRNA, mutant KH-dsRNA, and the quadruple complex are equilibrated after 20.0 ns of simulation in explicit solvent. The average RMSD is about 5 Å, respectively.

To study the stability of the dsRNA, C5’ variations for holo and apo states are monitored and shown in Figure 2. The C5’ variations of KH-dsRNA, mutant KH-dsRNA, and PAZ-dsRNA are significantly smaller than that of apo-dsRNA, especially in the 3′ and 5′ terminal regions of dsRNA. The C5’ variations of dsRNA in quadruple complex for most regions are also lower than those of apo-dsRNA. Surprisingly, the C5’ fluctuation of quadruple complex is obviously larger than that of apo-dsRNA in the loop region of bases 11–14. This suggests that the quadruple complex is relatively stable and the conformational change might focus on bases 11–14 for dsRNA. These conformational changes may play some roles in the translation upregulation of microRNA as will be discussed in the proposed pathway.

### 2. Binding Mode between dsRNA and Protein

To study the driving force for binding induced conformational change for dsRNA, the electrostatic, hydrophobic, and hydrogen-binding interactions between dsRNA and KH/PAZ were analyzed.

**Figure 3:**
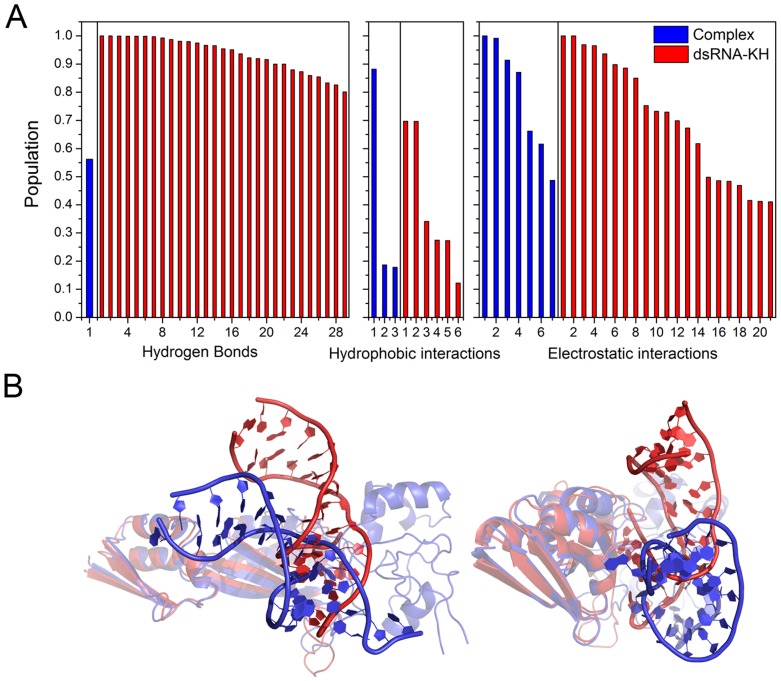
Interaction and alignment of structure for KH-dsRNA and quadruple complex. A: Hydrogen bond, hydrophobic, and electrostatic interactions between KH domain and dsRNA for KH-dsRNA and quadruple complex, blue for complex and red for dsRNA-KH. Significant differences for interactions indicate different binding modes. B: The alignment of dsRNA-KH and quadruple complex, blue for complex and red for dsRNA-KH.

Figure 3A shows the electrostatic, hydrophobic and hydrogen-binding interactions between dsRNA and KH. 29 hydrogen bonds are found for KH-dsRNA with population higher than 50% and just one hydrogen bond for the quadruple complex. However, there is not common hydrogen bond for these systems. Therefore, KH domain might adjust the conformation of dsRNA and change the binding mode with dsRNA in the quadruple complex. To further understand the conformational changes, the average structures of KH-dsRNA and the quadruple complex were aligned and shown in Figure 3B. The orientation of dsRNA was different for two complexes. The interactions were focused on 3′-end of mRNA and 5′-end of microRNA for the quadruple complex. Two stable hydrophobic interactions can be found: Ile318/U15 and Val293/A14 for KH-dsRNA, and one hydrophobic contact of Ile318/A14 for the quadruple complex, with populations higher than 50%. Among these hydrophobic interactions, there is one conserved and marginally stable hydrophobic contact (Ile304/A14) with a population around 20%. The mutational experiment shows that Ile304Asn in the KH domain causes a particularly severe case of mental retardation in patient. [21] This suggests that Ile304 is the key residue for dsRNA binding. This will be further analyzed below. For electrostatic interactions, there are 14 and 6 electrostatic interactions between positively charged amino acids and the phosphates of dsRNA for KH-dsRNA and the quadruple complex, respectively, with populations higher than 50%. There are two conserved interactions with common positive charged residues of Arg315 and Arg317 providing electrostatic interacts with the phosphates of the dsRNA. The difference of interactions between dsRNA and KH confirms that the binding mode might have been significantly changed. Hydrogen bonding, hydrophobic, and electrostatic interactions between PAZ and dsRNA for PAZ-dsRNA and quadruple complex are shown in Figure S2A. The interactions between PAZ and dsRNA also decrease upon the binding of KH domain, especially for the hydrogen bond. However, the magnitudes of changes are not significant as those between KH and dsRNA upon PAZ domain’s binding. Alignment of average structures for these two complexes was also shown in Figure S2B. The orientation of dsRNA is similar to each other for two systems.

**Figure 4:**
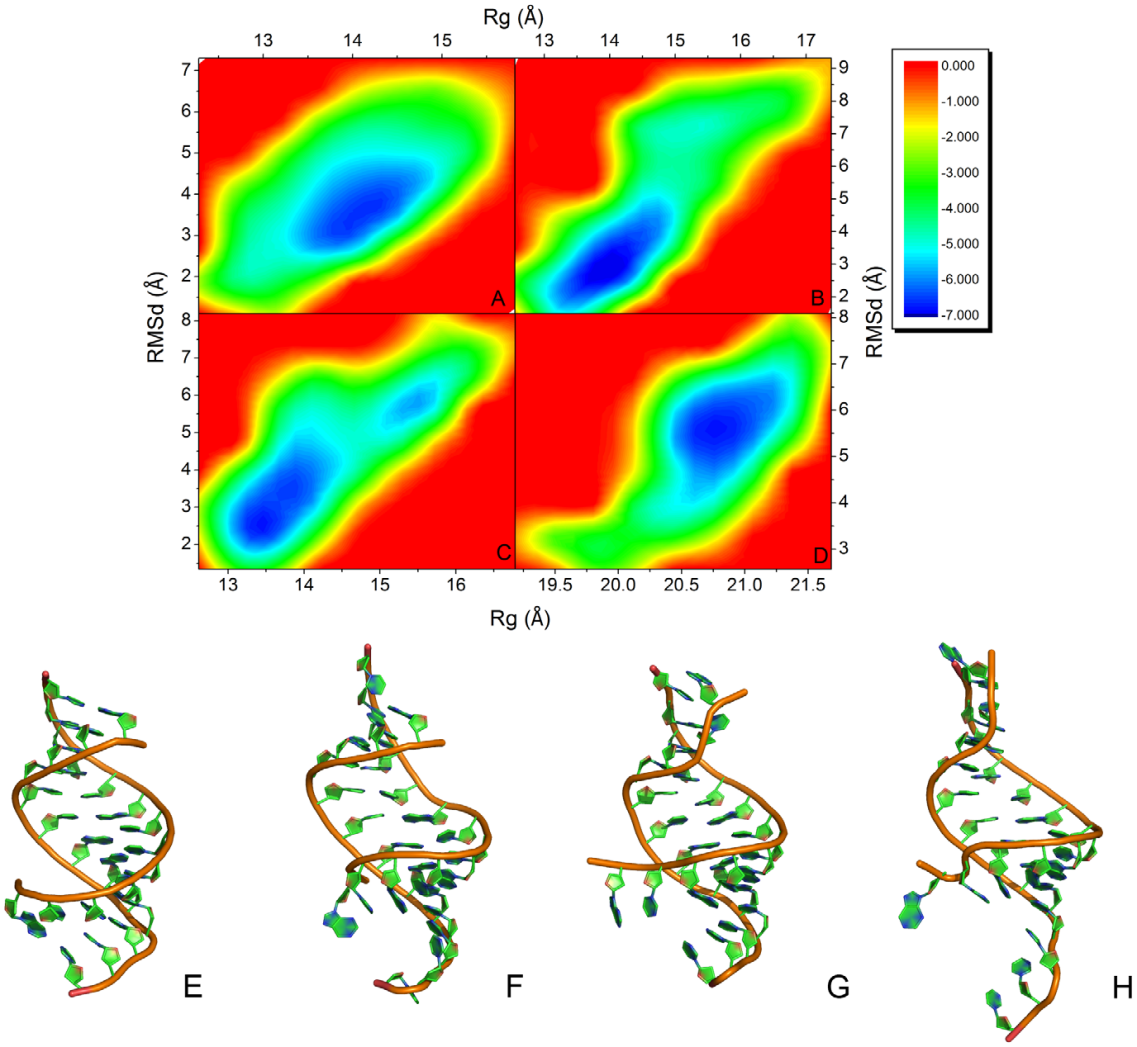
Energy landscape with the variables of RMSD and Rg and average structure for dsRNA. A: apo-dsRNA; B: PAZ-dsRNA; C: KH-dsRNA; D: complex; E: average structure of dsRNA for apo-dsRNA; F: average structure of dsRNA for PAZ-dsRNA; G: average structure of dsRNA for KH-dsRNA; H: average structure of dsRNA for complex.

To explore the conformational adjustment of dsRNA upon binding of PAZ and KH, the energy landscapes with the reaction coordinates of C5’ RMSD and the radius of gyration (*Rg*) of dsRNA were analyzed and shown in Figure 4. The analysis indicates the existence of a major free energy basin for apo-dsRNA with *Rg* values between 13.5 Å and 14.5 Å and C5’ RMSD between 3.0 Å and 5.0 Å. For PAZ-dsRNA, there is a free energy basin for dsRNA with *Rg* between 13.8 Å and 14.3 Å and C5’ RMSD between 2.5 Å and 3.5 Å. Furthermore, there is also a free energy basin for KH-dsRNA with *Rg* between 13.3 Å and 14.0 Å and C5’ RMSD between 2.0 Å and 4.0 Å. This suggests that the conformation of dsRNA has the propensity of contraction upon the binding of PAZ or KH domain, respectively. For the quadruple complex, one free energy basin is also found with *Rg* between 14.5 Å and 15.8 Å and C5’ RMSD between 4.5 Å and 6.5 Å. The average structures of dsRNA for four systems are also shown in Figure 4. The figure indicates that the double strand of dsRNA for the quadruple complex has the propensity of elongation and opening. The energy landscapes with the reaction coordinates of Cα RMSD and *Rg* for PAZ and KH were analyzed and shown in Figure S3. For PAZ, there is a free energy basin with *Rg* between 14.0 Å and 14.4 Å and Cα RMSD between 2.1 Å and 2.7 Å in PAZ-dsRNA. Then the free energy basin for PAZ changes to *Rg* between 18.0 Å and 18.5 Å and Cα RMSD between 2.7 Å and 3.3 Å in quadruple complex. The similar result can be found for KH domain. This suggests that PAZ and KH also adjust their conformations upon the dsRNA binding.

**Figure 5:**
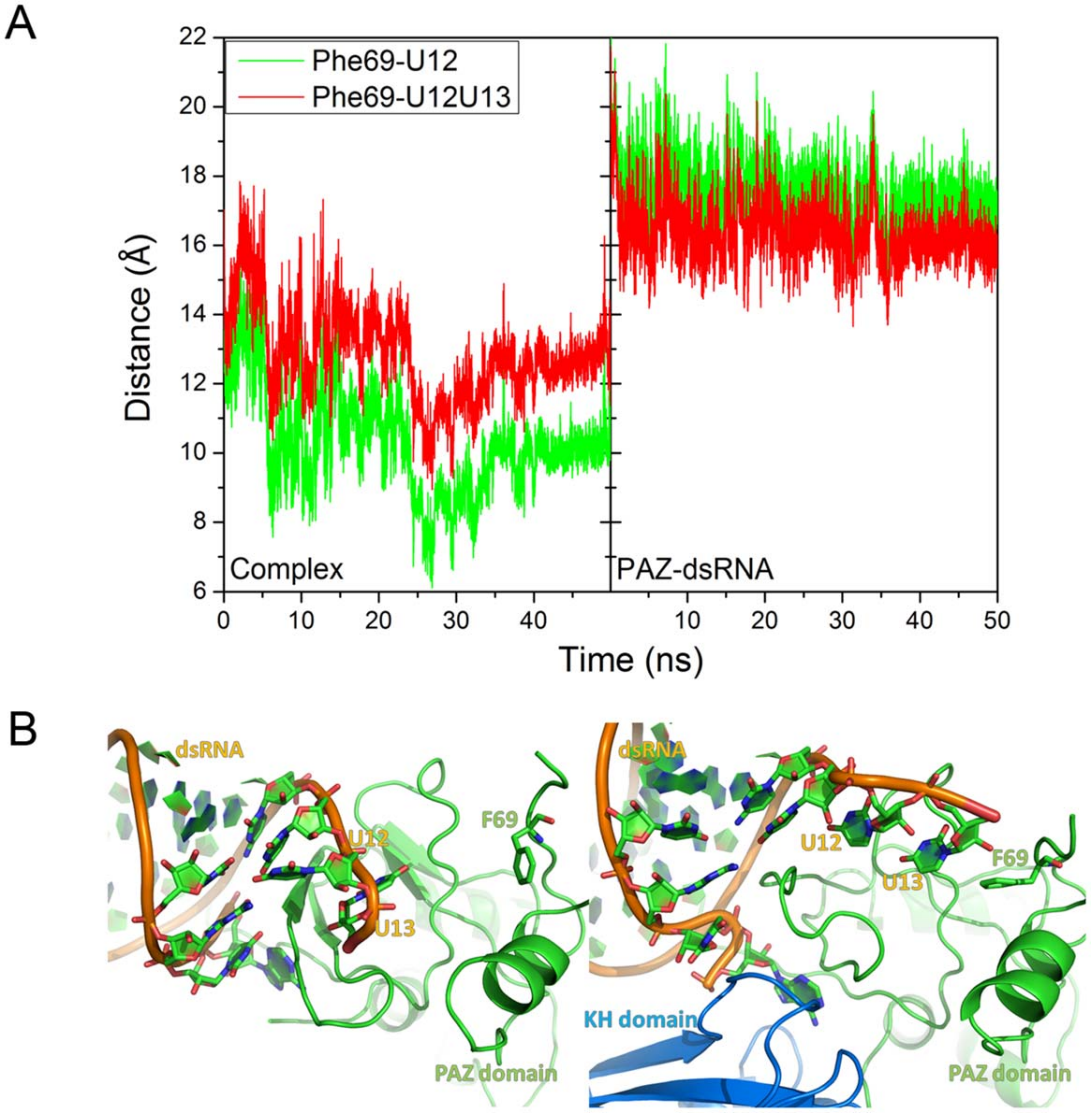
Distance between Phe69 of PAZ and U12 or U12U13 of dsRNA and average structure of PAZ-dsRNA and quadruqle complex. A: Distance between Phe69 of PAZ and the base of 3′ terminal of mRNA, U13 in green and U12/U13 in red, respectively. B: The detailed structure near U12/U13 and Phe 69.

Previous work reports that Phe69 of PAZ domain has hydrophobic interactions with the base of 3′-terminal. [22] The distances were calculated between Phe69 and the mass center of base for U12 and U12U13 for PAZ-dsRNA and quadruple complex to confirm this finding (Figure 5A). For PAZ-dsRNA, the distances were about 18.0 Å between Phe69 and the mass center of U12 or U12U13 and kept constant. In the quadruple complex, the corresponding distances are about 12.0 Å and with propensity of decrease. Figure 5B visualizes this change between two complexes at the same viewpoint. In PAZ-dsRNA complex, dsRNA’s backbone keeps its canonical structure and U12/U13 keep far away from Phe69. While in quadruple complex, PAZ domain extrudes dsRNA so that U12 and U13 got closer to Phe69. This suggests that dsRNA partially enter the cavity of PAZ domain upon binding of the KH domain. That is, KH-binding induces a conformational change of dsRNA, consistent with the previous work. [23]

**Figure 6:**
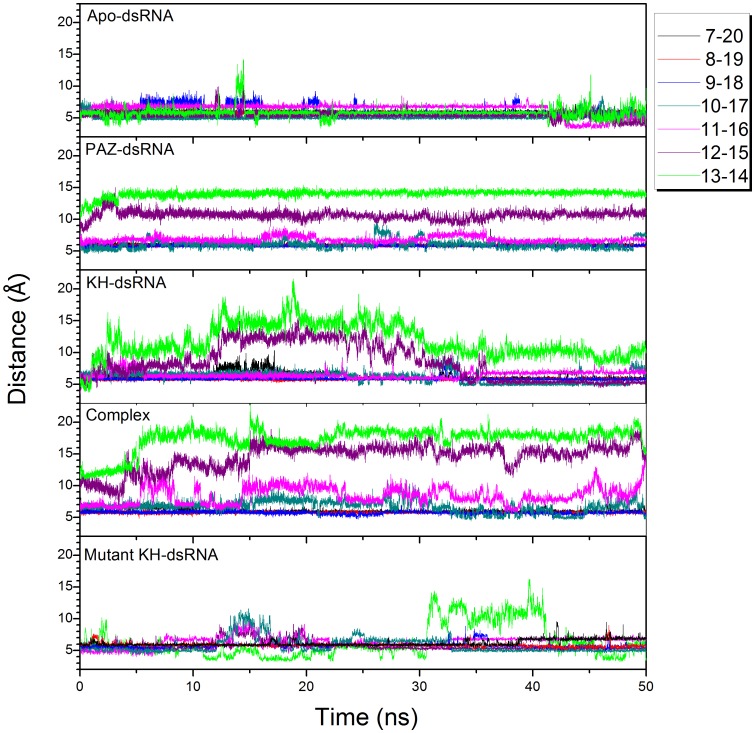
Distance between the base of mRNA and mircoRNA for apo-dsRNA, PAZ-dsRNA, KH-dsRNA, complex, and mutant KH-dsRNA.

To explore conformational changes of dsRNA, the distance of each base pair between mRNA and miR369-3 is monitored and shown in Figure 6. There is no significant change for apo-dsRNA and the distance is about 5∼6 Å for its 13 base pairs during 50.0 ns simulation. For PAZ-dsRNA, the distance for base pair 13–14 increases from 10 Å to 12 Å and that for base pair 12–15 increases from 9 Å to 10 Å. The distances of other base pairs do not change noticeably. For KH-dsRNA, the distances of base pair 13–14 and 12–15 have large fluctuation until 35 ns simulation, and then their values are smaller than 9 Å. As for the quadruple complex, the distance increases from 10 Å to 18 Å for base pair 13–14 and from 10 Å to 16 Å for base pair 12–15 during 50 ns simulation. Interestingly, the distance of base pair 11–16 also increases from 7 Å to 12 Å at the end of 50 ns simulation. This suggests that the double strand of dsRNA in the quadruple complex gradually opens according to the order of base pair from 5′-terminal of microRNA.

**Figure 7:**
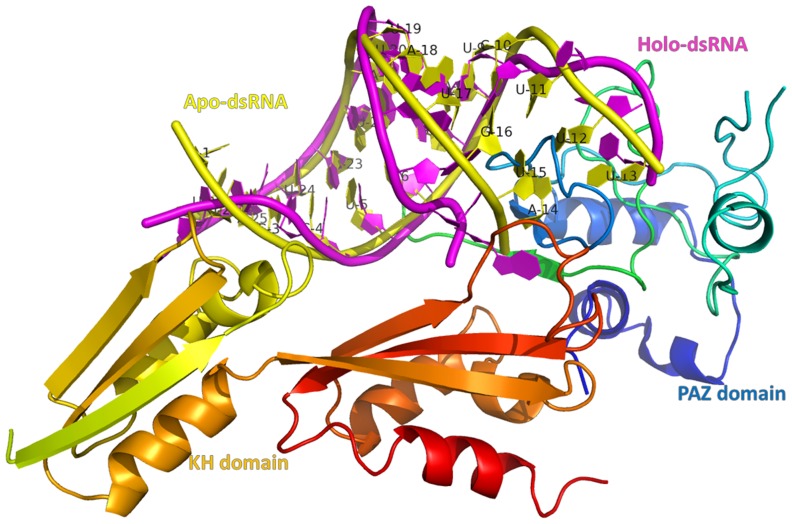
Alignment between apo-dsRNA and the quadruple complex. The number of base pairs is marked.

The distances for six base pairs from 3′-terminal of microRNA are shown in Figure S4. For dsRNA, KH-dsRNA, and the quadruple complex, the distance of these base pairs is about 6 Å and dsRNA is in dynamic equilibrium. The distances of two base pairs for PAZ-dsRNA increase at the beginning of 40.0 ns, then decrease at the end of 60.0 ns. The landscape of distance difference for base pairs between the quadruple complex and apo-dsRNA is shown in Figure S5. The landscape can reflect the relative conformational change of dsRNA between holo and apo states. The deep red area shows that the distance differences between bases 13–14, 12–15, and 11–16 are positive values. These bases correspond to 5′-terminal of microRNA and 3′-terminal of mRNA, indicating that the double strand significantly opened upon PAZ and KH binding. This is consistent with the distance analysis. The alignment between average structures of apo-dsRNA and holo dsRNA in the quadruple complex is shown in Figure 7. At 3′-end of mRNA and 5′-end of microRNA in apo-dsRNA (yellow), base pairs keep native conformation, while holo dsRNA (magenta) loses native contacts at its terminal, suggesting that the opening of double strands for dsRNA at 3′-terminal of mRNA and 5′-terminal of microRNA is significant.

Our simulation shows that the binding of PAZ or FXR1 stabilizes microRNA-mRNA. In the quadruple complex, KH domain changes the binding mode with dsRNA. Furthermore, the combination of AGO2 and FXR1 may facilitate the strand separation of dsRNA. The base pair distance changes are consistent with the results of conformational analysis presented in the previous section. As will be shown below, the strand separation of dsRNA is the key step for translation upregulation of microRNA and will be further discussed below.

### 3. Mutation Analysis

Experimental point mutation shows that Ile304Asn causes a particularly severe case of mental retardation. [24] To understand its molecular mechanism, we conducted *in silico* mutation Ile304Asn and subject the mutant to the same explicit-solvent molecular dynamics simulation up to 50.0 ns. The electrostatic and hydrogen bonding interactions between mutant KH and dsRNA are shown in Figure S6. There are 12 stable electrostatic interactions for mutant KH-dsRNA with population higher than 50%. These values are less than those of wild type KH-dsRNA. Comparing with WT KH-dsRNA, no stable hydrogen bond was found between mutant KH and dsRNA with population higher than 50%. Furthermore, hydrophobic interactions are lost between mutant KH and dsRNA. Indeed our predicted binding free energy lost is about 8 kcal/mol with −26.1 kcal/mol for WT and −17.9 kcal/mol for mutant using the MMPBSA method. [25] The contribution of binding free energy for each residue and base is listed in Figure S7. The lowest binding free energy is focused on the region of Ile304. This suggests that Ile304 is the key residue for the stability of KH-dsRNA and consistent with the previous mutant experiment. [24]

**Figure 8:**
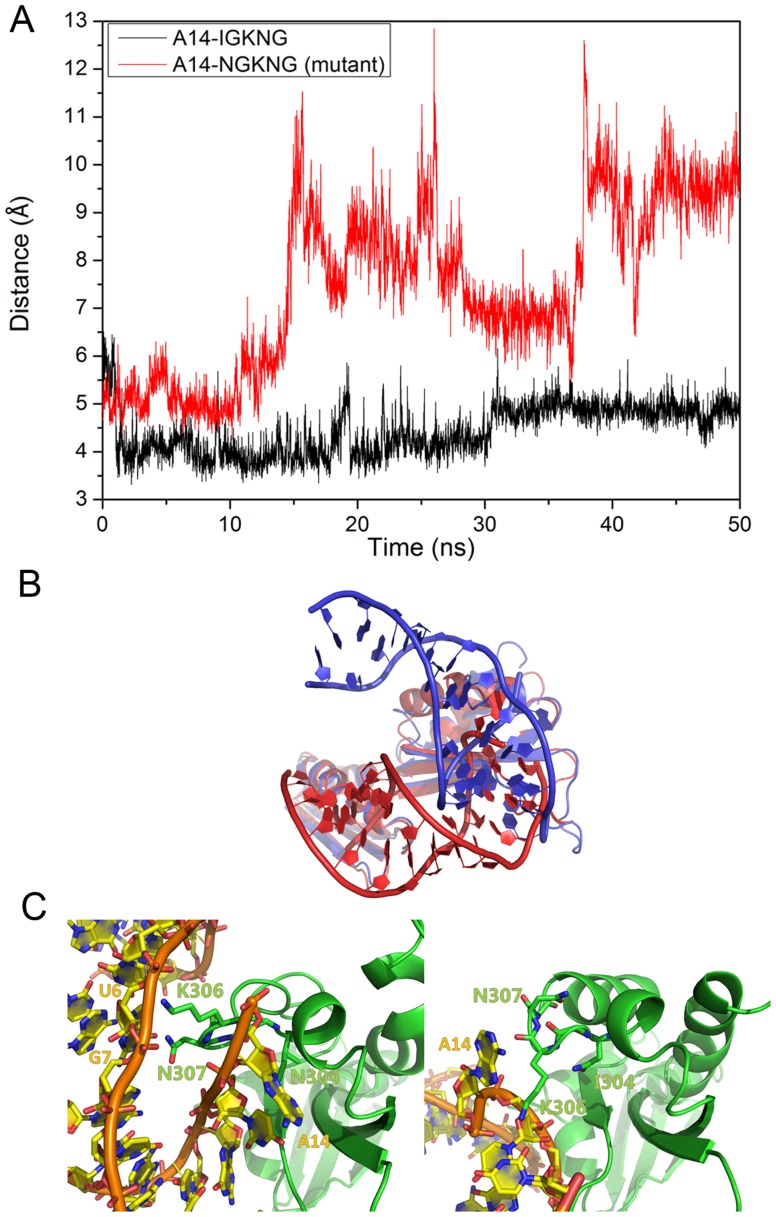
Distance between GXXG motif of KH and A14 of microRNA and structure alignment for WT and mutant KH-dsRNA. A: Distance between A14 of microRNA and I(N)GKNG motif of KH for WT and mutant KH-dsRNA. B: KH-domain based alignment of WT (blue) and mutant (red) dsRNA-KH complex. C: The detailed interaction between GXXG motif and dsRNA. I/N304–308 is shown in green stick.

## Discussion

### 1. Comparison with Experiment

The structural analysis suggests that Ile304 of KH is the critical residue in stabilizing the complex. [24] The simulation of mutant Ile304Asn suggests that the mutant completely abolishes all hydrophobic and eight electrostatic interactions between dsRNA bases and KH domain. The secondary structures for WT and mutant KH domain are shown in Figure S8. Comparison with WT, the secondary structure of mutant KH around Ile304 changes to β bridge from hydrogen bond turn. This is in agreement with the mutational experiment that Ile304Asn results in loss of secondary structure of KH domain and caused a particularly severe case of mental retardation in one patient. [21], [24], [26].

**Figure 9:**
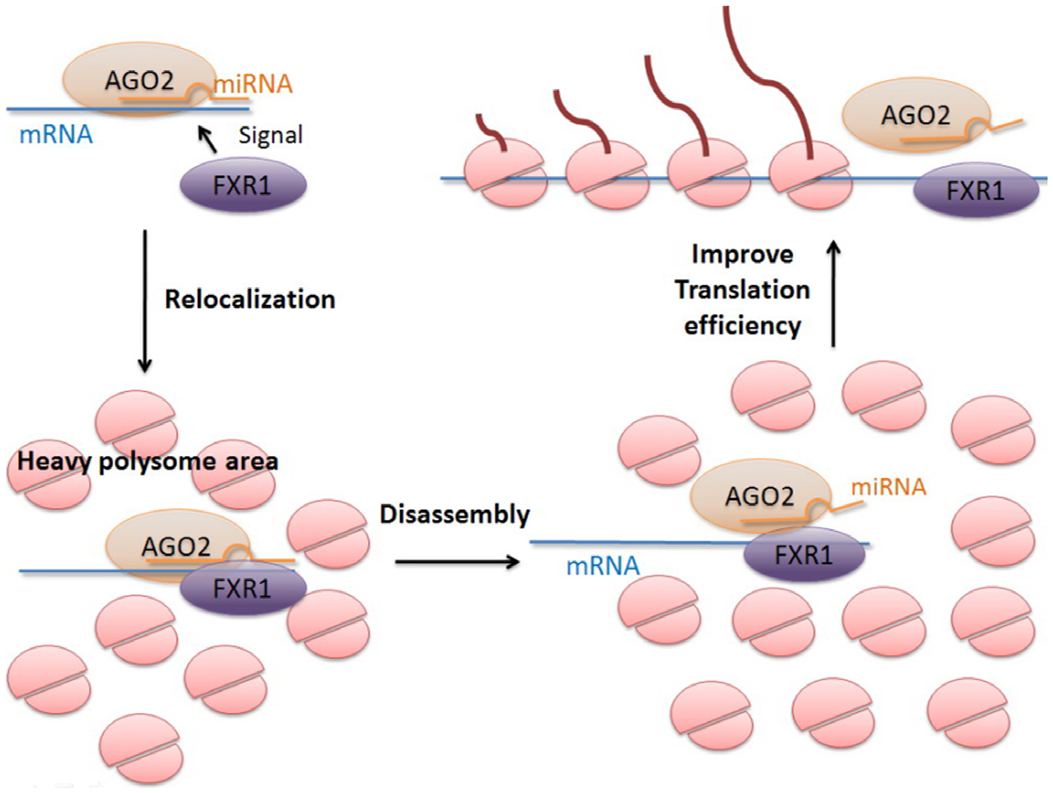
microRNA up-regulation translation mechanism.

Furthermore, sequence alignment and evolution analysis suggest that the GXXG is an invariant motif in KH domain and this conserved motif is crucial for nucleic acid binding. [21], [26], [27], [28] In order to confirm the interaction between the GXXG motif of KH domain and the corresponding base of dsRNA, the distances between the GXXG motif of KH and A14 of microRNA for WT and mutant KH-dsRNA are shown in Figure 8A. For KH-dsRNA, the distances tend to decrease and can form hydrophobic interactions. For the mutant, the distance increases sharply at 15.0 ns, then stays at a constant value of 9 Å. The hydrophobic interaction between mutant Asn and the GXXG motif is lost. The alignment of WT and mutant dsRNA-KH are shown in Figure 8B. Similar to PAZ-dsRNA, the orientation of dsRNA for mutant dsRNA-KH is also different from WT dsRNA-KH, indicating functional changes after the mutation.

Electrostatic interactions, such as Lys306/U6, Lys306/A14, and Lys306/G7, are observed in our simulation. Besides, there is also one marginally stable hydrogen bond (Asn307/A14) with a population around 40%. Here, Lys306 and Asn307 are the residues of the GXXG motif. Therefore, although homology modelling structure with the very high sequence identity has the limitation, simulation result is consistent with the observation of x-ray experiment. [21] Stable interactions could be found between dsRNA and I304-G308 (GXXG motif) in WT dsRNA-KH complex, while few interactions were found within this region for mutant complex.

Finally, our simulation suggests that the interactions with the PAZ and KH domain lead to conformational change in dsRNA and eventually lead to its strands to separate. This is consistent with the experimental observation that FXR1 can exchange miRNA/miRNA* strands. [16]

### 2. Molecular Mechanism of Upregulation Translation

As reviewed in Introduction, knock down and immunofluorescence experiments suggest that AGO2 and FXR1 proteins are required in mammalian G0-induced activation [8] and mRNAs recruit a translation activating AGO2-FXR1 complex, which relocalizes to polysomes in G0. [11] These valuable experimental findings offered several isolated events during the transcription regulation pathway of microRNA. However a complete picture of the regulation pathway is still missing. Given our additional molecular dynamics simulation analyses, a possible regulation mechanism of microRNA may be proposed as shown in Figure 9. There are potentially four steps in the microRNA upregulated translation of mRNA. At step 1, FXR1 is recruited to assemble miRNA:mRNA and AGO2 complex under the condition of serum starvation. [11], [29] Our simulation also indicates the existence of a stable quadruple complex of PAZ-dsRNA-KH. At step 2, the complex is relocalized to polysomes based on a previous experimental observation. [11] At step 3, FXR1 and AGO2 might facilitate the disassemble of mRNA from the miRNA:mRNA duplex. Indeed, our simulation suggests that the interactions with the PAZ and KH domain lead to conformational change in dsRNA and eventually lead to its strands’ separation. Time evolution for the fraction of native binding contacts (Qb) between mRNA and microRNA for quadruple complex is shown in Figure S9. The strand opening process is quite rapid with a half time (τ) of 4.96 ns. Indeed, the observations are consistent with Figures 5 and 6 that the first few base pairs are easiest and first to break out. This step might be a key one for microRNA upregulation. At step 4, the polysomes may improve the translation efficiency of mRNA. [30] Experimental observations also show that mRNAs actively being translated are associated with polysomes and an increased polysome association improves the translation efficiency. [30] The mechanism proposed here helps further studies on the microRNA-dependent upregulation of translation. The proposed mechanism can also be validated by further x-ray structural analyses of AGO2/FXR1 complexes in quiescent (G0) mammalian cell lines.

## Methods

### 1. Molecular Modeling

The dsRNA of mtARE *vs* miRmt369-3 was retrieved from the literature. [8] NAB package was used to build dsRNA. [31] The crystal structure of KH domain of FXR1 has not been released. Fortunately, isofomer a of FXR1 has 81% sequence identification with the whole protein of FMRP (pdb code: 2qnd). [32] Based on this template, structure of KH domain of FXR1 was constructed using SWISS-model with default set. The constructed structure was not minimized. The high reliability of this modeling server when the template shares a high identification has been evaluated by several literatures. [32], [33], [34] The next task is to assemble them into quadruple complex with appropriate relative position according to the binding site between RNA and proteins. [26], [35] The GXXG motif of KH domain can bind the loop of microRNA. [21] Binding mode for PAZ domain and dsRNA has been clarified and crystal structure for this complex has been released (pdb code 1SI2) [35]. Based on this structure, we manually constructed the quadruple complex according to GXXG motif of KH domain and the loop of microRNA. The quadruple complex was constructed and shown in Figure 1. The stability of these systems can be validated by MD simulation.

### 2. Molecular Dynamics Simulation

The atomic coordinates of dsRNA, PAZ-dsRNA, KH-dsRNA, and PAZ-dsRNA-KH were obtained from molecular modeling. Point mutant was modeled with SCWRL3. [36] Hydrogen atoms were added using the LEAP module of AMBER8. [37] Counter-ions were used to maintain system neutrality. All systems were solvated in a truncated octahedron box of TIP3P [38] waters with a buffer of 10 Å. Particle Mesh Ewald (PME) [39] was employed to treat long-range electrostatic interactions with the default setting in AMBER8. The parm99 force field was used for the intramolecular interactions. [40] The SHAKE algorithm [41] was used to constrain bonds involving hydrogen atoms. 1000-step steepest descent minimization was performed to relieve any structural clash in the solvated systems. This was followed by heating up and brief equilibration for 20 ps in the NVT ensemble at 298K with PMEMD of AMBER8. Langevin dynamics with a time step of 2 fs was used in the heating and equilibration runs with a friction constant of 1 ps^−1^. To study the kinetics of each solvated system, 50.0 ns each (60.0 ns for PAZ-dsRNA) in the NPT ensemble at 298K were simulated with PMEMD of AMBER8. A total of 260 ns trajectories were collected for five solvated systems (dsRNA, PAZ-dsRNA, KH-dsRNA, PAZ-dsRNA-KH, and mutant KH-dsRNA) at 298K, respectively, taking about 45,500 CPU hours on the in-house Xeon (1.86 GHz) cluster.

### 3. Data Analysis

Hydrophobic contact assignment was handled with in-house software. [42], [43], [44], [45], [46], [47], [48], [49], [50], [51] These residues and nucleotides are in contact when their center mass of side chains are closer than 6.5 Å for the complex. Electrostatic (i.e. charge-charge) interactions are assigned when the distance between the center mass of positive charge residue and the dsRNA phosphate backbone is less than 11 Å. [52] A previous study has shown that charge-charge interactions up to 11 Å were found to contribute to protein/RNA binding free energies. [52] The energy landscape was performed by calculating normalized probability from a histogram analysis and plotted with Origin 8.5. [53] For each simulation, sampling was conducted every 10 ps (5000 snapshots for 50 ns simulation and 6000 snapshots for 60 ns simulation). *Rg* and RMSD were both separated into 8 bins. The energy landscape was plotted among these 64 (8×8) bins. All the 3D molecular representations were shown with PyMOL 0.99rc6.

## Supporting Information

Figure S1 **C5’ RMSD of dsRNA for four systems.**
(TIF)

Figure S2 **Interaction between PAZ and dsRNA and alignment of PAZ-dsRNA and complex.** (A) Hydrogen bond, hydrophobic, and electrostatic interactions between PAZ and dsRNA for PAZ-dsRNA and complex. (B) Stereoscopical viewings of PAZ-domain based alignment of PAZ-dsRNA and quadruple complex, blue for complex and red for PAZ-dsRNA.
(TIF)

Figure S3 **Energy landscape with the variables of RMSD and Rg and average structure for PAZ and KH domain.** A: PAZ-dsRNA; B: PAZ in quadruple complex; C: KH-dsRNA; D: KH in quadruple complex; E: average structure of PAZ in PAZ-dsRNA; F: average structure of PAZ in complex; G: average structure of KH in KH-dsRNA; H: average structure of KH in complex.
(TIF)

Figure S4 **The distance between the base of mRNA and mircoRNA for apo-dsRNA, PAZ-dsRNA, KH-dsRNA, complex, and mutant KH-dsRNA.**
(TIF)

Figure S5 **The landscapes of distance difference for a pair of C5’ atoms in different bases between the ternary complex and apo-dsRNA.** Red regions represent positive value, blue regions for negative value.
(TIF)

Figure S6 **Hydrogen bond and electrostatic interaction for mutant KH-dsRNA.**
(TIF)

Figure S7 **The binding free energy of each residue and base for WT and mutant KH-dsrNA.**
(TIF)

Figure S8 **The secondary structure of KH domain for WT and mutant.** A: dsRNA-KH. B: dsRNA-mutant KH. Purple represents β sheet, blue for β bridge, cyan for 3_10_ helix, green for π helix, yellow for hydrogen bond turn, orange for α helix, red for bend.
(TIF)

Figure S9 **Kinetics fitting for the opening of dsRNA.** The red curve is fitted by single exponential function of Aexp(−t/τ)+B.
(TIF)
