# Supplementary figures and images for: Atomistic Mechanism of MicroRNA Translation Upregulation via Molecular Dynamics Simulations
Source: PLoS One. 2012 Aug 27;7(8):e43788. doi: 10.1371/journal.pone.0043788 (PMC3428290; doi:10.1371/journal.pone.0043788)

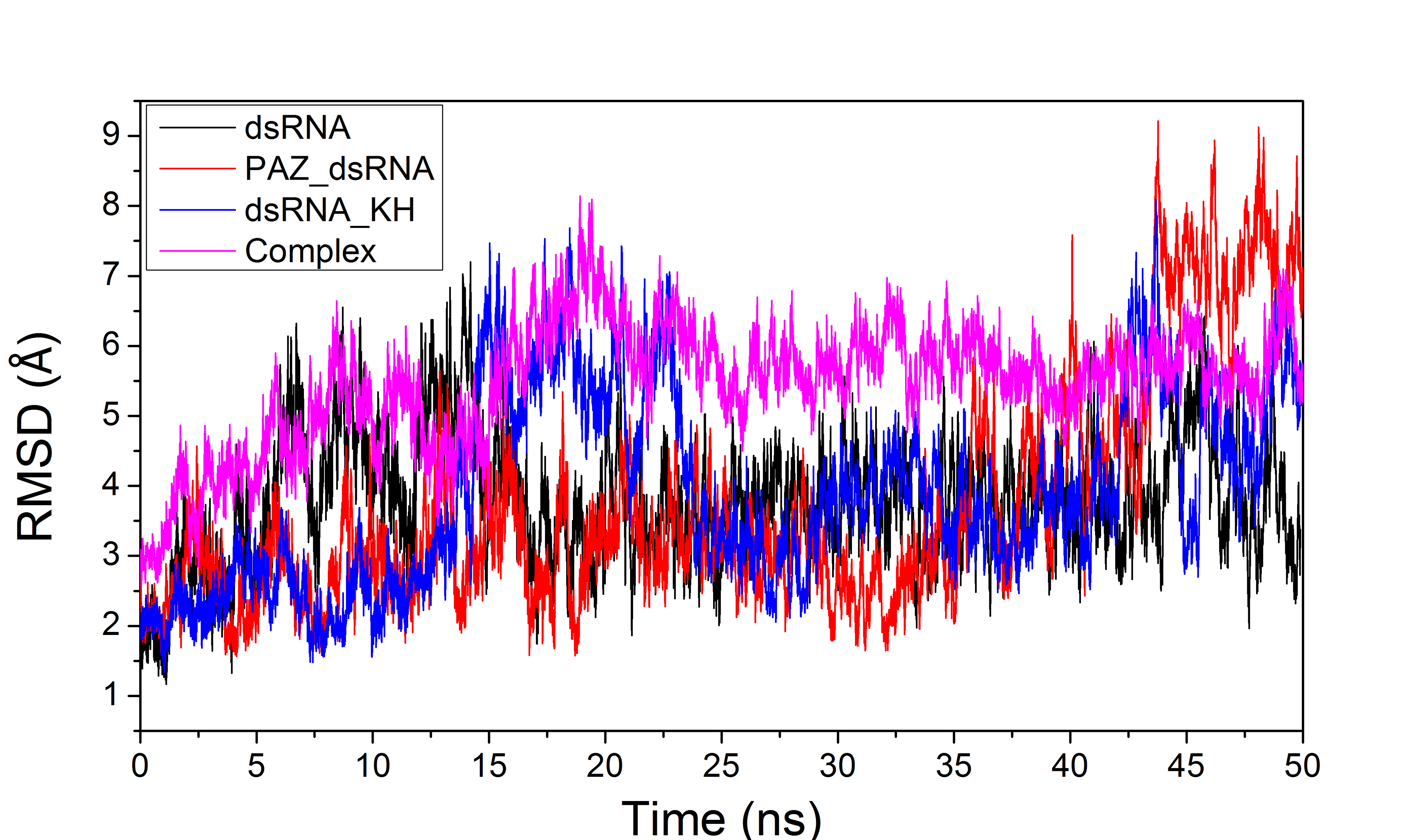

Supplement: Figure S1 — C5’ RMSD of dsRNA for four systems. (TIF) [file pone.0043788.s001.tif]

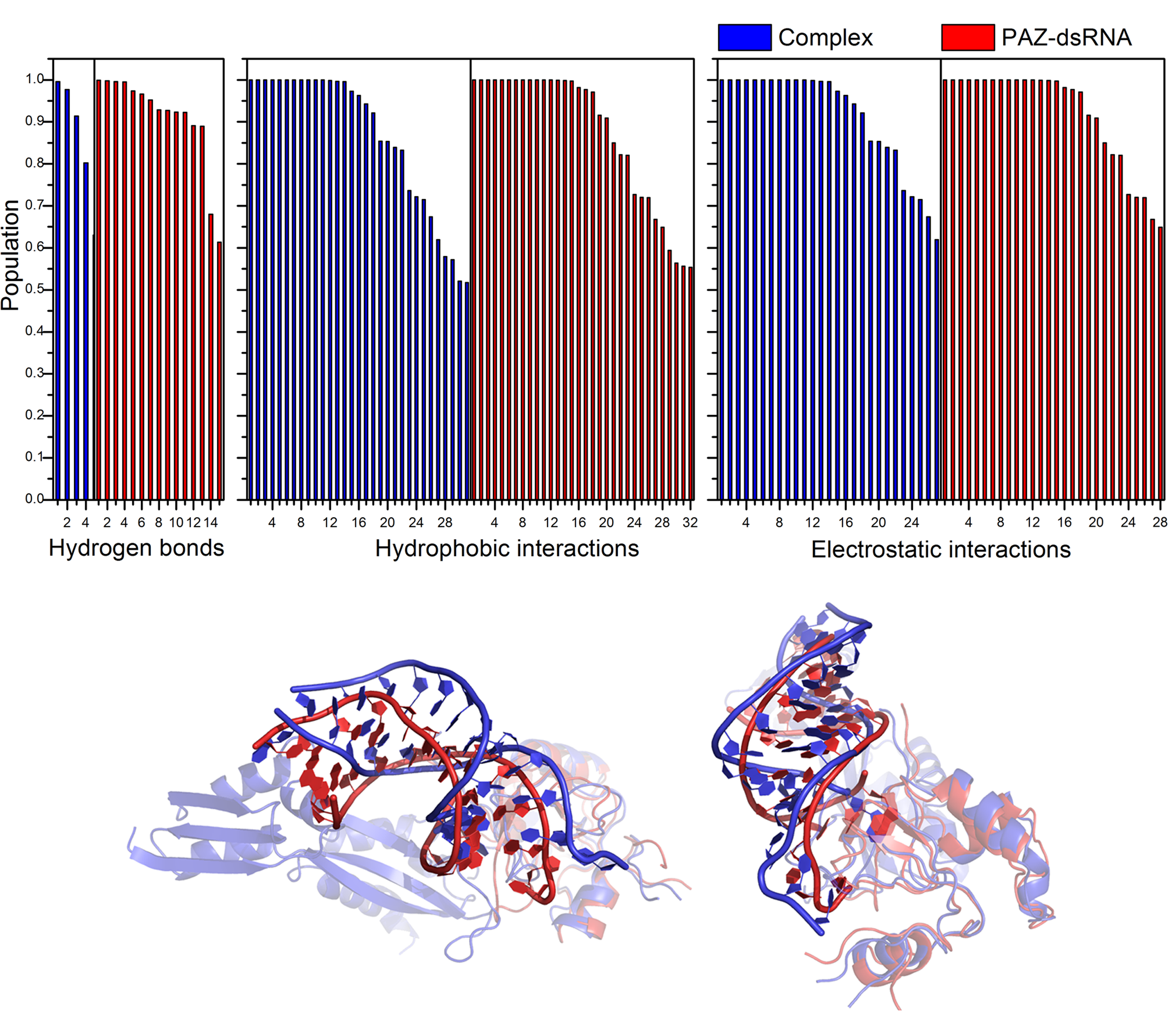

Supplement: Figure S2 — Interaction between PAZ and dsRNA and alignment of PAZ-dsRNA and complex. (A) Hydrogen bond, hydrophobic, and electrostatic interactions between PAZ and dsRNA for PAZ-dsRNA and complex. (B) Stereoscopical viewings of PAZ-domain based alignment of PAZ-dsRNA and quadruple complex, blue for complex and red for PAZ-dsRNA. (TIF) [file pone.0043788.s002.tif]

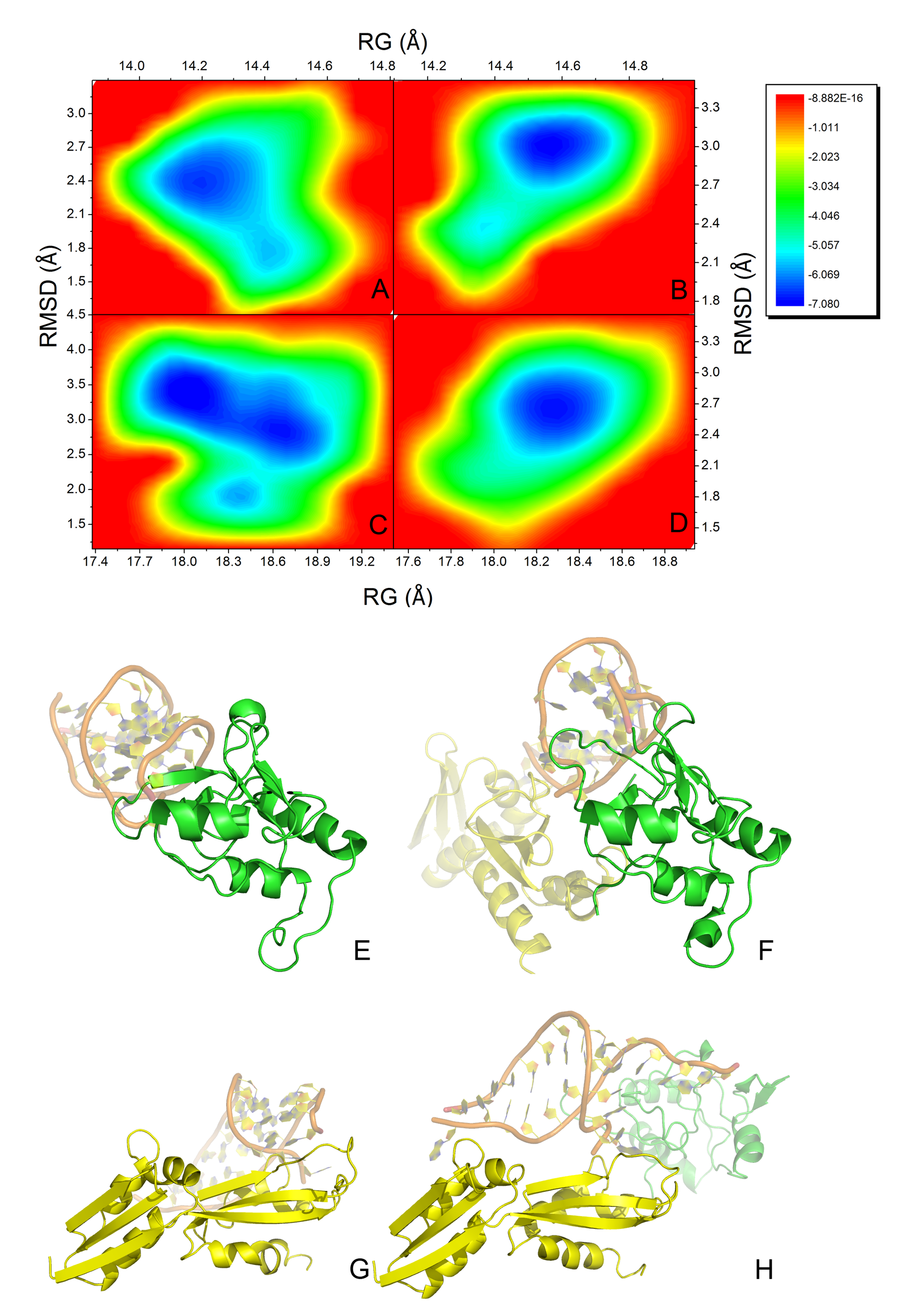

Supplement: Figure S3 — Energy landscape with the variables of RMSD and Rg and average structure for PAZ and KH domain. A: PAZ-dsRNA; B: PAZ in quadruple complex; C: KH-dsRNA; D: KH in quadruple complex; E: average structure of PAZ in PAZ-dsRNA; F: average structure of PAZ in complex; G: average structure of KH in KH-dsRNA; H: average structure of KH in complex. (TIF) [file pone.0043788.s003.tif]

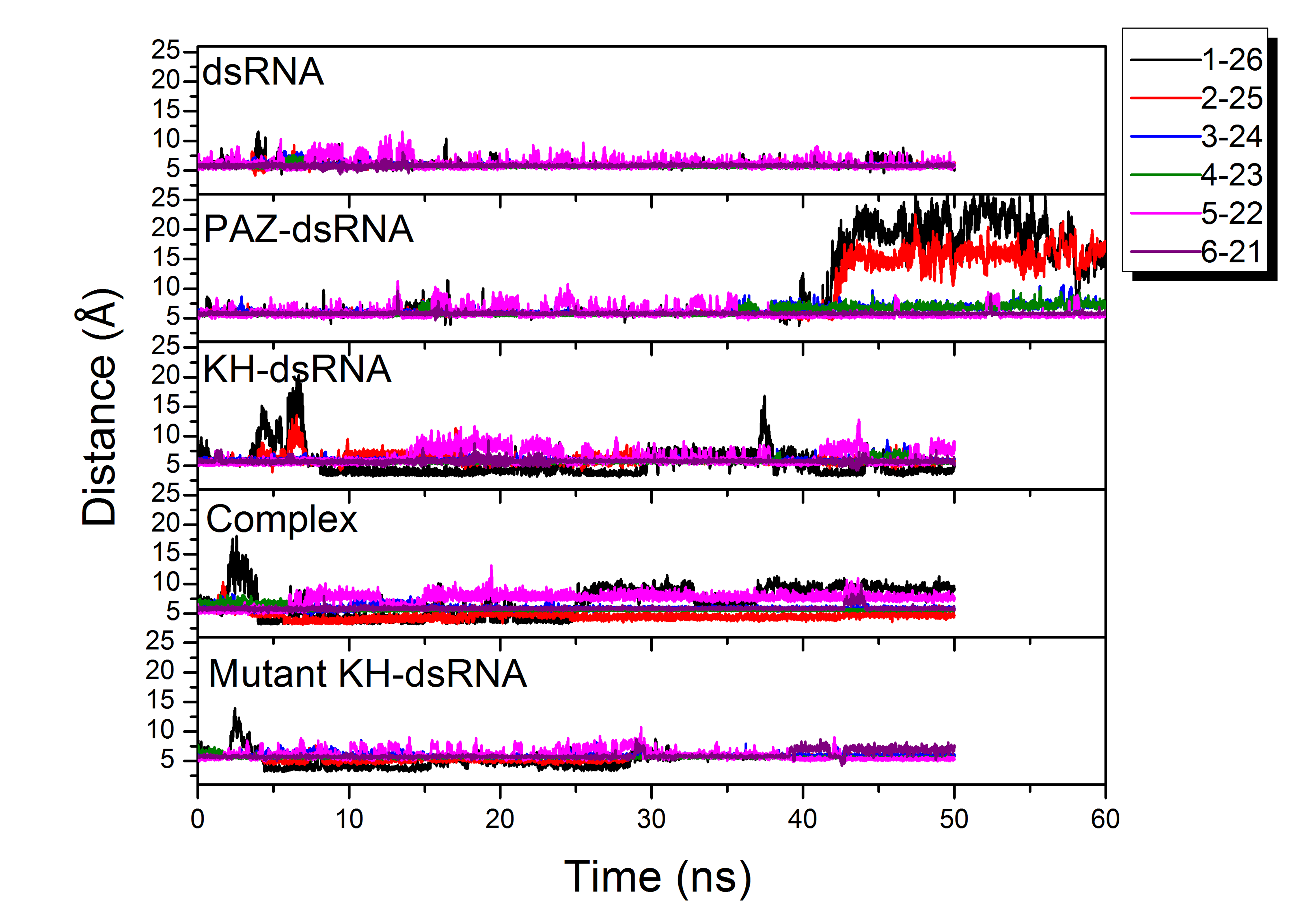

Supplement: Figure S4 — The distance between the base of mRNA and mircoRNA for apo-dsRNA, PAZ-dsRNA, KH-dsRNA, complex, and mutant KH-dsRNA. (TIF) [file pone.0043788.s004.tif]

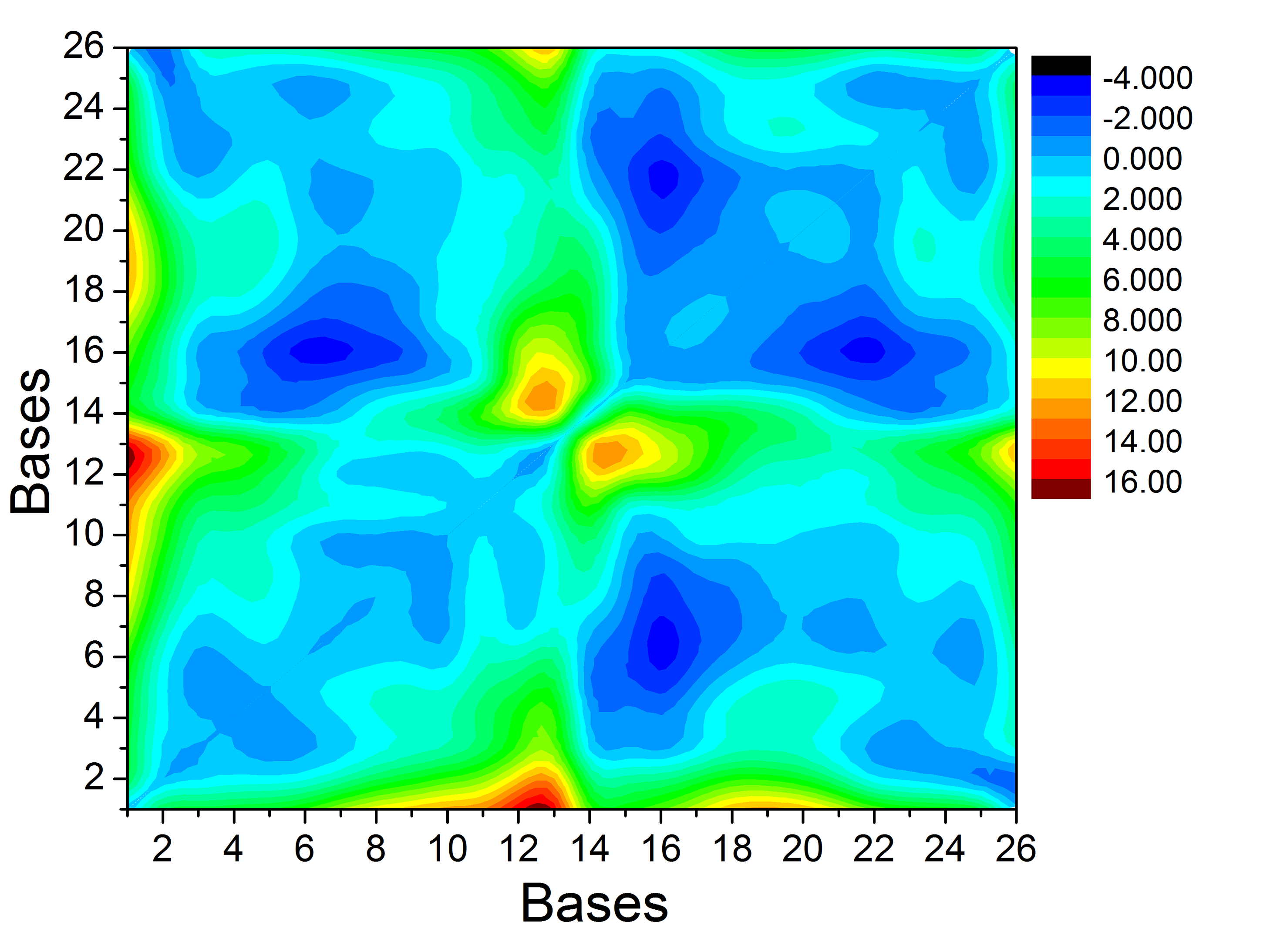

Supplement: Figure S5 — The landscapes of distance difference for a pair of C5’ atoms in different bases between the ternary complex and apo-dsRNA. Red regions represent positive value, blue regions for negative value. (TIF) [file pone.0043788.s005.tif]

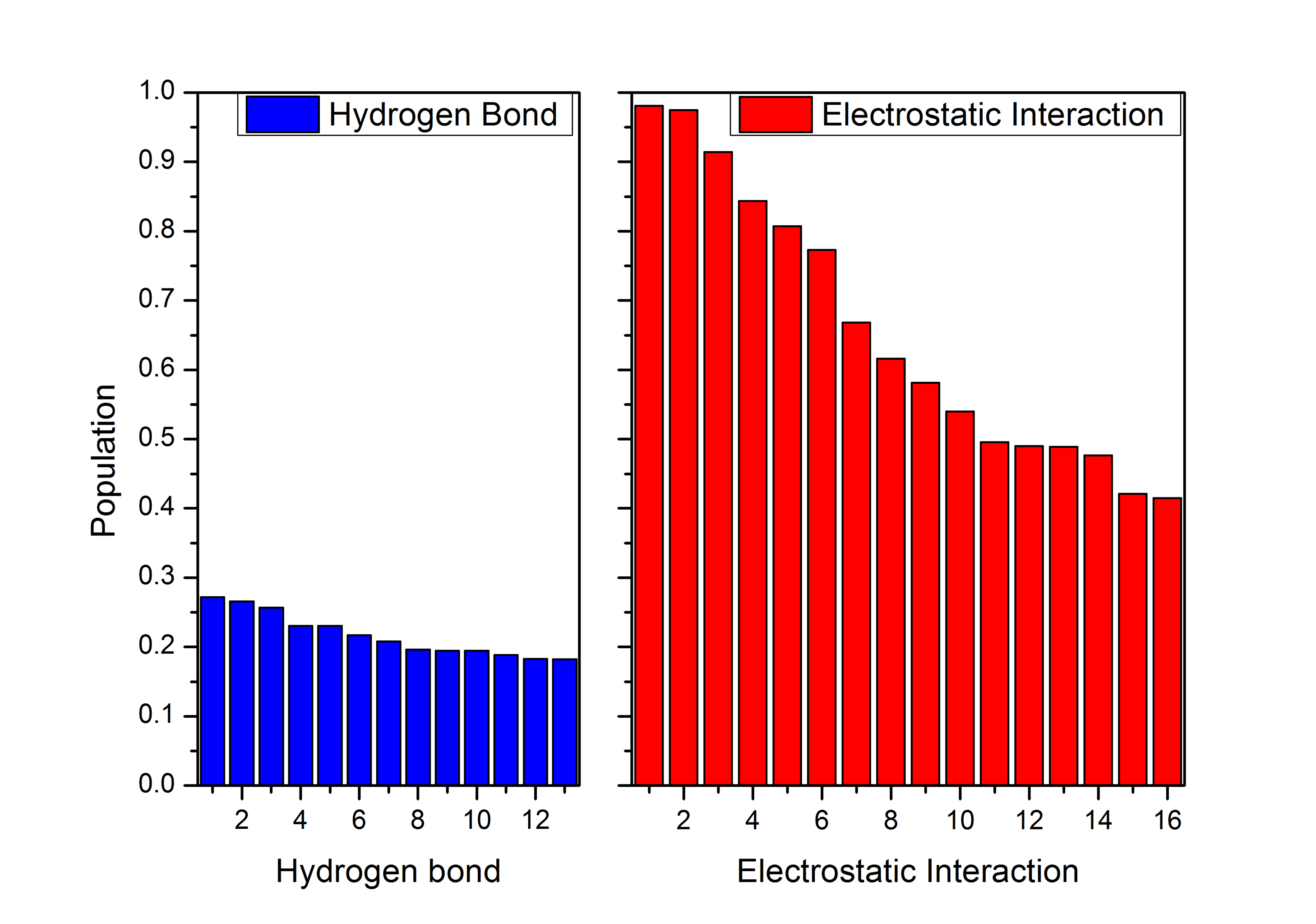

Supplement: Figure S6 — Hydrogen bond and electrostatic interaction for mutant KH-dsRNA. (TIF) [file pone.0043788.s006.tif]

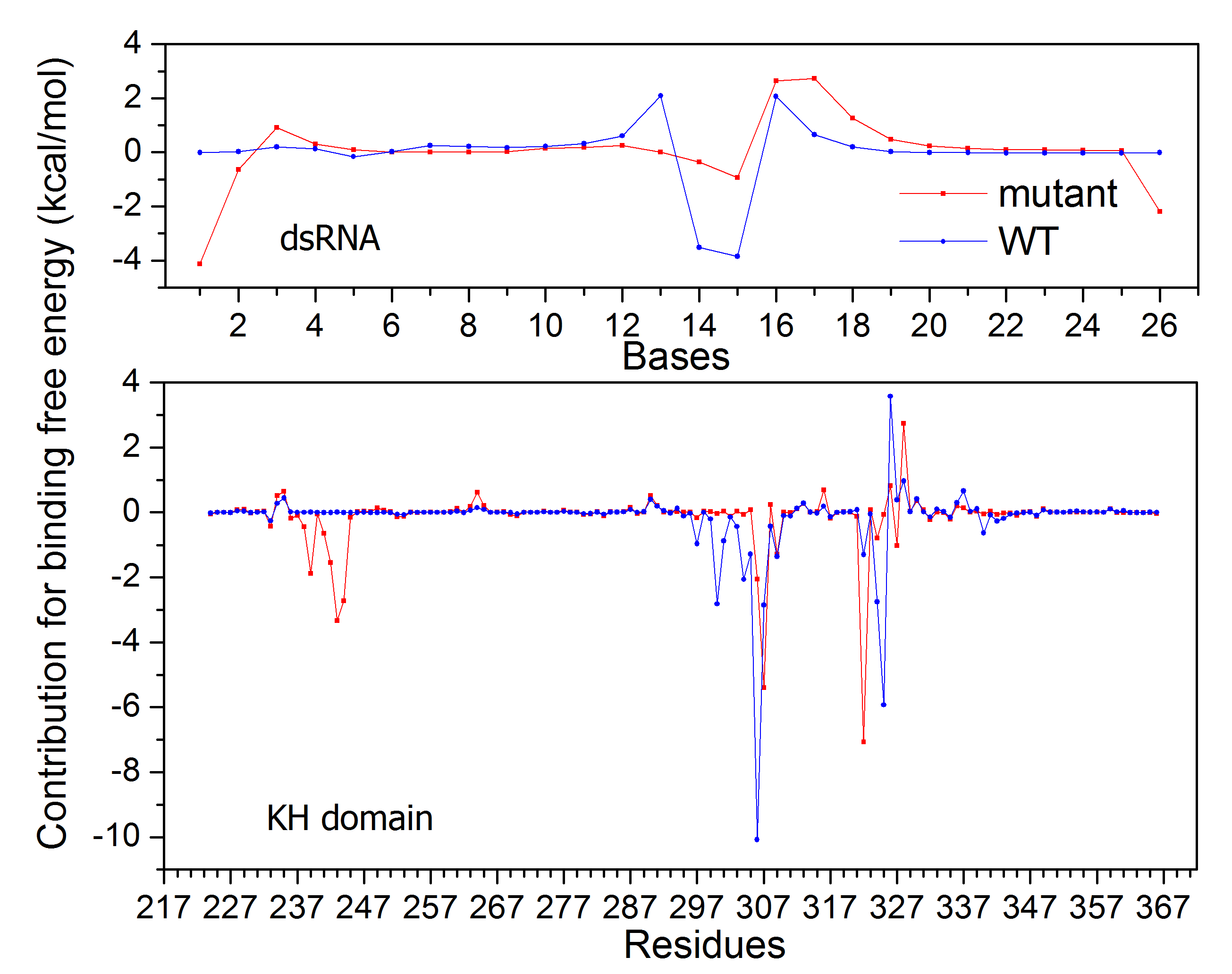

Supplement: Figure S7 — The binding free energy of each residue and base for WT and mutant KH-dsrNA. (TIF) [file pone.0043788.s007.tif]

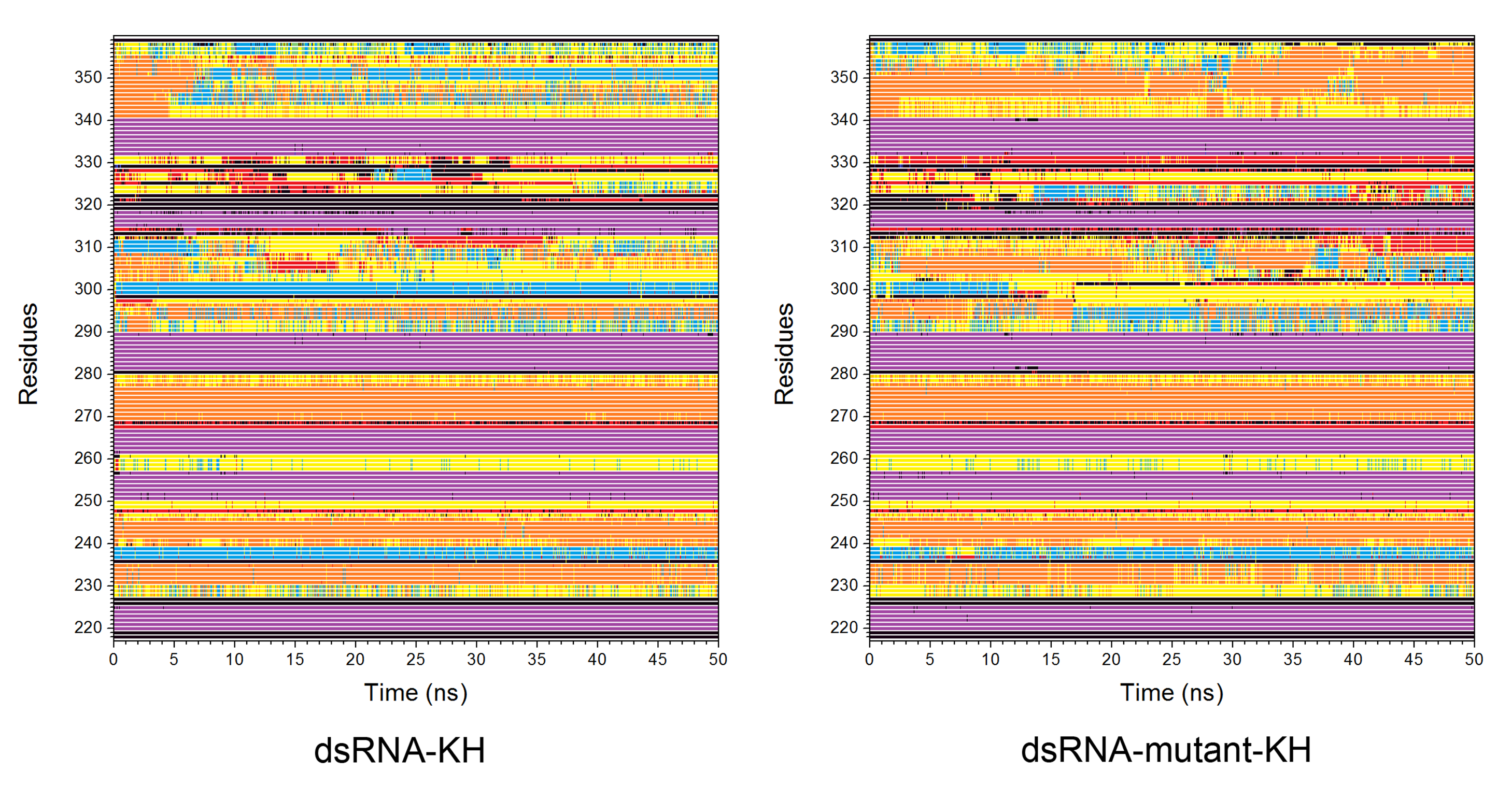

Supplement: Figure S8 — The secondary structure of KH domain for WT and mutant. A: dsRNA-KH. B: dsRNA-mutant KH. Purple represents β sheet, blue for β bridge, cyan for 310 helix, green for π helix, yellow for hydrogen bond turn, orange for α helix, red for bend. (TIF) [file pone.0043788.s008.tif]

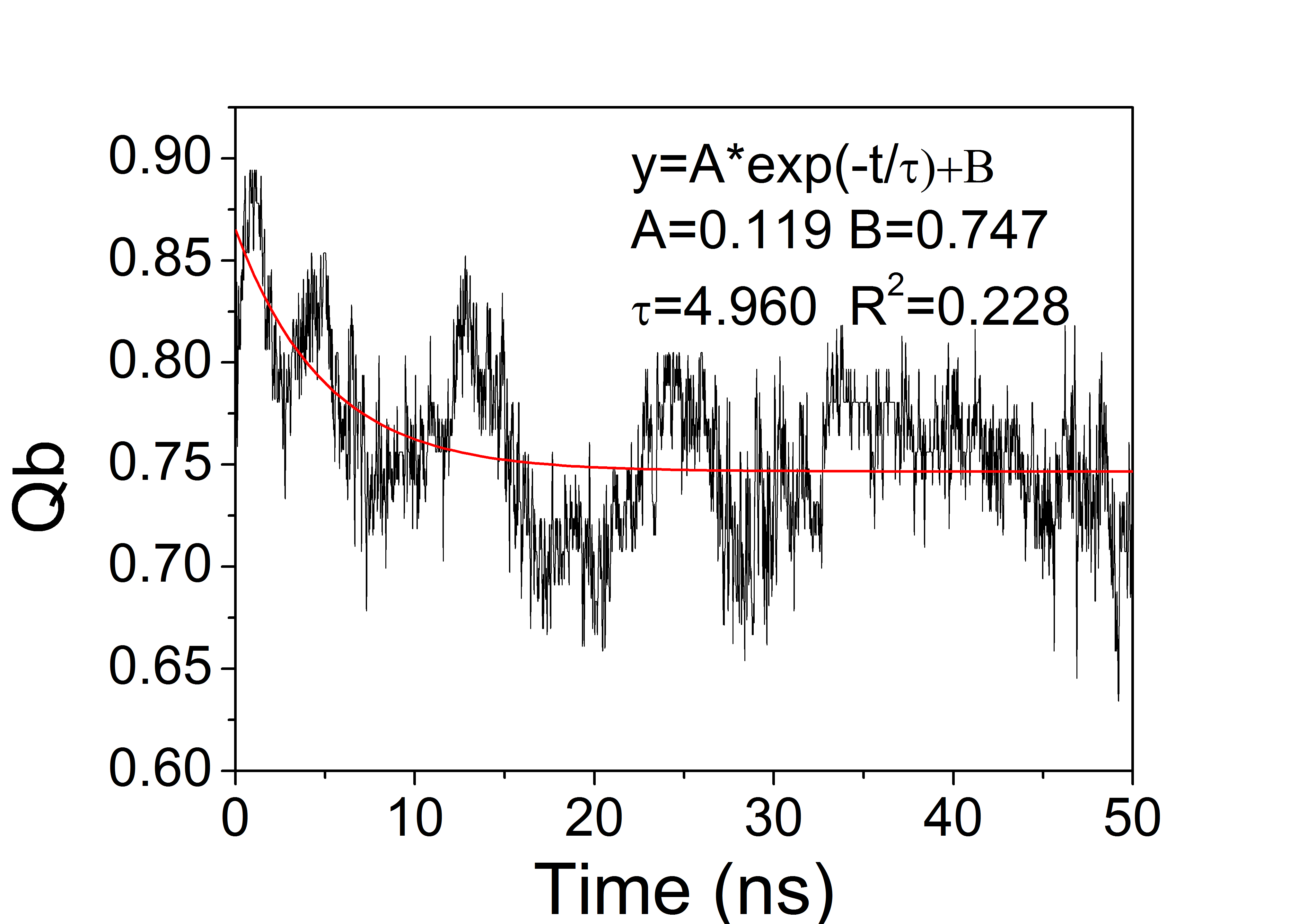

Supplement: Figure S9 — Kinetics fitting for the opening of dsRNA. The red curve is fitted by single exponential function of Aexp(−t/τ)+B. (TIF) [file pone.0043788.s009.tif]
